# Inhibition of miRNA associated with a disease-specific signature and secreted via extracellular vesicles of systemic lupus erythematosus patients suppresses target organ inflammation in a humanized mouse model

**DOI:** 10.3389/fimmu.2023.1090177

**Published:** 2024-06-13

**Authors:** Nicholas A. Young, Emily Schwarz, Braden M. Zeno, Shane Bruckner, Rosana A. Mesa, Kyle Jablonski, Lai-Chu Wu, Elisha D. O. Roberson, Wael N. Jarjour

**Affiliations:** ^1^ Division of Rheumatology and Immunology, Department of Internal Medicine, The Ohio State University Wexner Medical Center, Columbus, OH, United States; ^2^ Department of Medicine, Washington University School of Medicine, St. Louis, MO, United States; ^3^ Department of Biological Chemistry and Pharmacology, The Ohio State University, Columbus, OH, United States; ^4^ Department of Genetics, Washington University, St. Louis, MO, United States

**Keywords:** systemic lupus erythematosus, miRNA - microRNA - miR, Extracellular vesicle (EV), exosome (vesicle), Toll like recepotor - TLR7 - TLR8, locked nucleic acid (LNA), inflammation, autoimmunity

## Abstract

**Introduction:**

Distinct, disease-associated intracellular miRNA (miR) expression profiles have been observed in peripheral blood mononuclear cells (PBMCs) of systemic lupus erythematous (SLE) patients. Additionally, we have identified novel estrogenic responses in PBMCs from SLE patients and demonstrated that estrogen upregulates toll-like receptor (TLR)7 and TLR8 expression. TLR7 and TLR8 bind viral-derived single-stranded RNA to stimulate innate inflammatory responses, but recent studies have shown that miR-21, mir-29a, and miR-29b can also bind and activate these receptors when packaged and secreted in extracellular vesicles (EVs). The objective of this study was to evaluate the association of EV-encapsulated small RNA species in SLE and examine the therapeutic approach of miR inhibition in humanized mice.

**Methods:**

Plasma-derived EVs were isolated from SLE patients and quantified. RNA was then isolated and bulk RNA-sequencing reads were analyzed. Also, PBMCs from active SLE patients were injected into immunodeficient mice to produce chimeras. Prior to transfer, the PBMCs were incubated with liposomal EVs containing locked nucleic acid (LNA) antagonists to miR-21, mir-29a, and miR-29b. After three weeks, blood was collected for both immunophenotyping and cytokine analysis; tissue was harvested for histopathological examination.

**Results:**

EVs were significantly increased in the plasma of SLE patients and differentially expressed EV-derived small RNA profiles were detected compared to healthy controls, including miR-21, mir-29a, and miR-29b. LNA antagonists significantly reduced proinflammatory cytokines and histopathological infiltrates in the small intestine, liver, and kidney, as demonstrated by H&E-stained tissue sections and immunohistochemistry measuring human CD3.

**Discussion:**

These data demonstrate distinct EV-derived small RNA signatures representing SLE-associated biomarkers. Moreover, targeting upregulated EV-encapsulated miR signaling by antagonizing miRs that may bind to TLR7 and TLR8 reveals a novel therapeutic opportunity to suppress autoimmune-mediated inflammation and pathogenesis in SLE.

## 1 Introduction

Systemic lupus erythematous (SLE) is a systemic autoimmune disease associated with a myriad of genetic and epigenetic aberrations, environmental triggers, and hormonal influences that affects multiple organs and displays a significant female predominance during reproductive years (1). Although the adaptive immune response has been investigated extensively in SLE, recent studies suggest that innate immunity may also play a significant role in disease pathogenesis and progression. Despite decades of basic and clinical research investigating SLE, the complex disease pathogenesis remains to be conclusively elucidated and definitively predictive biomarkers of disease activity have yet to be identified. Consequently, patients with SLE have incomplete diagnostic testing available and have limited therapeutic options with targeted agents and biologics.

While extracellular vesicles (EVs) were first reported in the literature half a century ago, their function has only been recently characterized in the last decade. EVs are defined by the International Society of Extracellular Vesicles (ISEV) as membrane-enclosed bodies secreted by cells and can be divided into subpopulations based on size and cellular biogenesis (2). Exosomes are 50 - 150 nm in diameter and originate from multi-vesicular, intracellular bodies. This study focuses on exosomes, which have the ability to carry highly perishable biocargo in a protected environment once secreted by cells. These biocargo include microRNA (miR) and other small, RNA species. Exosomes facilitate biocargo delivery to a target cell, which provides an important method of cell-to-cell communication. Current evidence is mounting in cancer, infectious disease, and autoimmunity alike for an immunomodulatory role of exosome-derived miRs (2–4). Moreover, as evidence of diagnostic utility, exosome-associated miR expression patterns can be used to predict drug resistance for patients with multiple myeloma (5).

Small non-coding miRs are about 22 nucleotides in length and are present in animals, plants, and viruses (6–9). Many miRs have been identified hitherto and each has the potential to target dozens of mRNAs by complimentary binding (10–14). In this way, the primary, canonical function of miRs has been described as a destabilizer and degrader of mRNA to function as a translational repressor of targets, which ultimately leads to gene silencing (10). While several recent studies have identified distinct changes in miR expression patterns in SLE (15–18), the identification and validation of novel EV-derived miR signatures may facilitate and guide the next phase of discovery in lupus. Accordingly, studies examining extracellular miR expression and their potential use as a predictor of therapeutic response in lupus patients have been considered. However, a comprehensive profiling of exosome-associated miRs has never been adequately explored.

Recently, we have demonstrated that extracellular miRs are primarily detected in EVs from lupus patients and that EV-encapsulated miR-21 induces both toll-like receptor (TLR)8 and cytokine expression *in vitro* (19). Furthermore, we have previously shown that estrogen lowers the threshold of immune cell activation to a greater extent in females and leads to enhanced TLR7 and TLR8 expression (20). Both TLR7 and TLR8 are X-linked immune system receptors, expressed predominately in macrophages, and stimulate innate inflammatory responses by binding to pathogen-associated, single-stranded (ss)RNA viral sequences. Considering that TLRs serve as an interface between innate and adaptive immunity (21), characterization of this association may contribute to a better understanding of their role in SLE pathogenesis. In addition to the well-characterized pathway of TLR7 and TLR8 activation by binding to viral ssRNAs, recent oncology studies have identified specific miRs capable of activating these receptors when packaged and secreted in EVs (22). This compelling finding characterizes an additional, non-canonical pathway of functionality where EV-delivered miRs can mediate pro-inflammatory signaling in distant cells. While cancer cells have been shown to produce tremendous quantities of exosomes (23), information about EV production and content in the context of autoimmune disease is sparse. Therefore, EV payload and the subsequent downstream effects miR biocargo on cells still requires extensive investigation in autoimmune diseases, such as SLE, and could translate into clinical diagnostic tests and identify novel pharmacological targets.

This significant, yet unmet need in the SLE diagnostic and therapeutic armamentarium can potentially be addressed by comprehensive examination of bulk RNA species derived from exosomes. Our results characterize intercellular pathways propagated via exosome signaling that represent an opportunity to identify potential therapeutic targets and small RNA signatures that can be further evaluated as diagnostic biomarkers. Moreover, our results suggest that targeting upregulated EV-encapsulated miR signaling by antagonizing miRs that bind to TLR7 and TLR8 may be a novel therapeutic opportunity to suppress autoimmune-mediated inflammation and pathogenesis in SLE. Collectively, these novel inflammatory EV signaling mechanisms present an opportunity to gain invaluable insight into SLE pathogenesis. The elucidation of EV-based pathways by miR profiling can unveil targets that could be translated into long-overdue therapeutics or more sensitive diagnostic tests.

## 2 Materials and methods

### 2.1 Human samples and collection

Lupus patients were recruited from The Ohio State University Wexner Medical Center (OSUWMC) Lupus Clinic and healthy controls from the local community. Lupus patients met the revised criteria of the American College of Rheumatology for SLE (24). All participants in the study provided informed consent and the research was conducted under an IRB approved protocol at OSUWMC. Blood samples were collected into EDTA containing tubes. Plasma was isolated by centrifugation, aliquoted, and frozen at -80^°^C. PBMCs were isolated by density gradient centrifugation over Ficoll as previously described (25).

### 2.2 Hormone treatment

After PBMCs isolation, cells were cultured in hormone free conditions as previously described (25) using phenol red-free RPMI 1640 (Life Technologies) and 5% charcoal stripped fetal bovine serum (Life Technologies). After resting overnight, cells were treated with 10 nM of 17β-estradiol (E2; Sigma-Aldrich) and collected after a 36 hr incubation according to previously established protocols (20).

### 2.3 RNA isolation and RT-qPCR

Cellular RNA was isolated, quantitated, synthesized to cDNA, and used for quantitative (q)PCR following previously described methods [14]. Briefly, RNA was isolated from PBMCs using the RNeasy Mini Kit (Qiagen Sciences) and from whole blood samples using the Paxgene Blood RNA Kit (PreAnalytix; Qiagen) according to manufacturer’s protocol. For analysis of RNA in EVs, total RNA was isolated according to manufacturer’s protocol using the MagMAX mirVana total RNA isolation kit (Thermo Fisher Scientific). All RNA isolations were quantitated using a NanoDrop 1000 spectrophotometer (NanoDrop Products) and were processed in the presence of chemical RNase inhibitor, RNAsecure (Thermo Fisher Scientific). cDNA was synthesized using the High Capacity cDNA Reverse Transcription Kit (Applied Biosystems) following manufacturer’s protocol and qPCR was performed using the TaqMan system (Applied Biosystems) with cDNA and gene-specific primers according to manufacturer’s protocol. All samples were run on the ABI Prism 7900HT Sequence Detection System (Applied Biosystems) and normalized to 18sRNA as an internal positive control. Results were analyzed using the 2^-ΔΔCt^ method (26).

### 2.4 Exosome/EV isolation and quantitation

Exosome/EV isolation was carried out using the Cold Spring Harbor ultracentrifugation protocol (27). Briefly, biospecimens (blood or urine) were centrifuged to remove any present cells and supernatants were then centrifuged to remove any remaining cell debris. The resulting supernatants were collected, transferred to new tubes, and centrifuged at 100,000 x g for 70 min at 4°C. The pelleted exosomes were resuspended in PBS, transferred to new centrifuge tubes, and centrifuged again at 100,000 x g for 70 min at 4°C to remove any contaminating protein aggregates that may have sedimented with the exosome pellet. The subsequent pellet containing purified exosomes was stored at -80°C prior to further processing. Murine EVs isolated from plasma were submitted directly to System Biosciences (SBI) for RNA isolation and miRNA profiling according to the Exo-NGS service, exosomal RNA sequencing protocol.

The NanoSight LM10 nanoparticle tracking analysis (NTA) system was used to measure the rate of Brownian motion of particles to determine the size and concentration of EVs according to manufacturer’s instructions (NanoSight). Briefly, samples were injected into the cubicle and the size of particles was obtained by determining the motion in fluid passing across the chamber. The particles/mL were measured and acquired from both experimental and control samples for comparison using area under the curve calculations obtained via the linear trapezoidal analysis method. Plasma was added to enzyme-linked immunosorbent assay (ELISA) ExoTEST plates (Hansa BioMed) according to manufacturer’s protocol and exosome concentrations were measured. This exosome quantitation method uses a human CD9 monoclonal antibody to capture and immobilize exosomes from various biological sources for quantitation.

### 2.5 RNA library preparation and analysis of high-throughput sequencing data (exosomes)

Following exosome isolation, RNA concentrations were determined using the Qubit high-sensitivity RNA assay and small RNA libraries were created using the Clontech SMARTer smRNA-Seq preparation kit according to manufacturer instructions (Takara). Small RNA inserts were further enriched using AMPure bead selection (Beckman Coulter) prior to quantifying with the Qubit DNA high-sensitivity kit. Equimolar amounts of each sample library were pooled to make a single collection for each sequencing run to limit batch effects. Libraries were sequenced on an Illumina HiSeq3000 with 50 base-pair, single-end reads. Raw sequencing read files were modified with cutadapt to remove sequencing adapters and non-template bases added during library preparation. miRs were counted by aligning (RNA-STAR) either to the whole GRCh38 human genome or the annotated human miRs (miRBase). Samples were irreversibly deidentified. Counts, differential expression, and regularized expression values (minimum required to reproduce the figures) can be found at: https://figshare.com/projects/2024_Jarjour_SLE_exosome_miRs/203685.

### 2.6 RNA library preparation and analysis of high-throughput sequencing data (PBMCs)

RNA was isolated from PBMCs according to manufacturer’s protocol using the RNeasy Mini Kit (Qiagen Sciences) after stimulation with estrogen in culture. Cell pellets were collected at 36 hrs and RNA sequencing for mRNA and miRNA transcriptional targets was performed through the Nucleic Acid Shared Resource (NASR) at OSUWMC. Targets with low counts were removed via edgeR::filterByExpr; this uses embedded filtering for sequencing depth and experimental design considerations. After filtering, 24,511 genomic targets were included in the mRNA analysis and 1,599 were included in the miRNA analysis, which correlates to 85% and 92% of the total, respectively. Normalization was done through the trimmed mean of M-values method and indicated that the filtering was adequate for downstream comparative evaluation. Using these data, results were normalized to untreated values for each subject individually to account for subject-to-subject variability in the datasets. Ingenuity^®^ pathway analysis (IPA) software was then used to filter the genes associated with estrogen treatment compared to untreated controls and selected for those known to be associated with immunological disorders or immune function, as defined by Ingenuity IPA Systems and according to our previously established analysis methods (20).

### 2.7 Mice and sample size determination

NOD.Cg*-Prkdcscid Il2rgtm1Wjl*/SzJ (NSG), NOD.Cg-*Rag1^tm1Mom^ Il2rg^tm1Wjl^*/SzJ (NRG), and NZM2410 mice were obtained from The Jackson Laboratories. Mouse maintenance and protocol 2017A00000032 was approved by the Institutional Animal Care and Use Committee at OSUWMC with adherence and recognition of ARRIVE guidelines. The animal facility was maintained at 22-23°C and between 30-50% relative humidity with a 12-hour light/dark cycle. Chow and water were available ad libitum. Blood urea nitrogen (BUN) levels and weights were measured biweekly in NZM2410 mice. As clinical indicators of kidney damage, experimental removal criteria was defined by a threshold of 20% weight loss and a BUN level above 50 mg/dL, as characterized previously (28). The sample sizes were based on calculations using reference data from previous studies with type one error of 0.05 and power of 0.8. To verify reproducibility and rigor of the data, experiments were repeated in accordance with NIH reproducibility guidelines. The number of mice used in each experiment was designed to provide a statistically significant result with a minimum number of animals. Because of possible sexual dimorphism for immune response, male and female mice were studied separately.

### 2.8 Adoptive transfers

Freshly isolated human PBMC preparations were washed in PBS and counted using a hemocytometer with trypan blue to ensure cell viability. All samples were kept separate and not pooled before injections. Prior to adoptive transfer into mice, PBMCs were treated with 100 nM total of human miRCURY (Exiqon) locked nucleic acid (LNA) inhibitors or equi-molar negative control (scrambled LNA): miR antagonists included miR21 CAACATCAGTCTGATAAGCT; miR29a AACCGATTTCAGATGGTGCT; and miR29b ACTGATTTCAAATGGTGCT. LNA-anti-miRs were combined with DOTAP liposomal transfection reagent according to manufacturer’s protocol and incubated approximately 10 hrs. PBMCs were injected intraperitoneally into 8-week old NSG or NRG mice (5.0 x 10^6^ cells/mouse); at least 2-3 mice were injected from each individual human sample. Mice were monitored every other day, including weights and physical signs of disease progression, and sacrificed three weeks after adoptive transfer for blood and tissue collection as described below.

### 2.9 Cytokine measurement

Murine serum was prepared from whole blood collected via subclavian artery at the experimental endpoint. Serum samples were analyzed by ELISA using the V-PLEX Pro-inflammatory Panel 1 mouse kit (Meso Scale Diagnostics) according to the manufacturer’s protocol and data was analyzed using Microsoft Excel (v2016), as previously described (28). All reported results were above the limit of detection (LOD) of the assay.

### 2.10 Tissue collection and histopathology

Tissues were resected from each mouse and flash frozen in liquid nitrogen followed by cryosectioning or immediately fixed by immersion in neutral buffered 10% formalin and then processed into paraffin (FFPE). Serial histological sections were stained with H&E (Leica Microsystems) following the manufacturer’s protocol, or labeled by immunohistochemistry (IHC) as detailed previously (28). Histological slides were digitally scanned for downstream quantitative image analysis.

H&E-stained FFPE tissue sections were subjected to blinded histopathological analysis using the 10x objective of a bright-field light microscope with predefined criteria (28, 29). Scanned slide images were analyzed by Aperio ImageScope digital analysis software (v9.1) as detailed previously to determine positive staining and lymphocyte localization by IHC (29, 30). Specifically, Aperio’s positive pixel count algorithm was run to quantify the extent of positive staining and lymphocyte localization using calibrated hue, saturation, and intensity values following previously described methods of computer-assisted image analysis (31). To define a mean positive pixel intensity value for each IHC slide, 10 measurements of identical total surface area of tissue were quantitated. All digital analysis was confirmed by manual slide interpretations using the 10x objective of a bright-field light microscope.

### 2.11 Flow cytometry

Blood was collected from chimeric mice by submandibular bleeding and leukocytes were purified for flow cytometry using ammonium chloride solution for red blood cell lysis. Cells were subsequently plated and blocked with anti-mouse FcR antibody (αCD16/CD32, BioLegend) in FACS buffer (PBS with 2% BSA and 1mM EDTA). Cells were then surface-stained with antibodies for anti-human CD3 (eBioscience), CD4 (Immunotech), CD8 (Caltag Laboratories), CD20 (eBioscience), CD14 (eBioscience), or CD56 (eBioscience) following manufacturer’s protocol. Data were collected on the BD LSRII Flow Cytometer (BD Biosciences) platform and exported for analysis via FlowJo (v.9.0; Treestar, Inc).

### 2.12 Statistics

Statistical analysis was performed by two-way ANOVA followed by Geisser-Greenhouse correction (Graph Pad Prism software v8.3.0) or by paired, two-tailed, Student *t*-tests (Microsoft Excel v2016). All numerical datasets were expressed as mean values with standard error of the mean (SEM) indicated using Graph Pad Prism or as mean values ± standard deviation using Microsoft Excel. Data was considered statistically significant if p ≤ 0.05.

## 3 Results

### 3.1 RNA expression profiles from extracellular vesicles are specific to biological sources and differentially regulated with lupus nephritis in human and murine samples

To evaluate whether enhanced exosome production and/or differential miR content within exosomes can contribute to the heightened inflammatory state observed in SLE, we isolated EVs from the plasma or urine of healthy controls or active lupus nephritis (LN) patients. Exosome isolation was carried out using the differential ultracentrifugation protocol, as outlined in the Cold Spring Harbor protocol (27, 32). This method remains a gold-standard in exosome studies and has been approved as a valid methodology by the ISEV (2). Following EV isolation, the NTA system was used to measure EV size and concentration. While initial plasma input contained EVs of various size, the ultracentrifugation isolation protocol yielded purified EVs consistent with exosome size, 100-200 nm diameter **(Supplementary Figure 1A**). Subsequently, small RNAs were quantitated via Agilent bioanalyzer and normalized to a starting concentration prior to RNAseq. The content of these exosomes demonstrated that they are indeed enriched for RNA species corresponding to typical miR size, approximately 22 nucleotides in length (**Supplementary Figures 1B, C**). To further characterize our EV isolations and confirm presence of exosomes, RNA concentration was measured by bioanalyzer analysis with increasing plasma volumes and demonstrated a trend of larger RNA yields with increased experimental input (**Supplementary Figure 2A**). Furthermore, nanoparticle tracking analysis of plasma-derived EV isolations yielded uniform histograms with a peak/average particle size of approximately 110 nm, which is within predicted exosome size range (**Supplementary Figure 2B**). To evaluate the EV isolations for exosome biomarker expression, western blotting for CD63 was performed on plasma isolations with increasing ultracentrifugation times. Longer ultracentrifugation yielded more total protein in isolations, which correlated with increased detection of CD63 (**Supplementary Figure 2C**). Following EV isolation, each sample was eluted and small RNAseq of at least 1 ng total RNA input was performed. Using the current version of miRbase, even at shallow sequencing depth and without further insert size optimization, we identified at least 94 known human miRs with at least 5 reads in all samples (data not shown).

**Figure 1:**
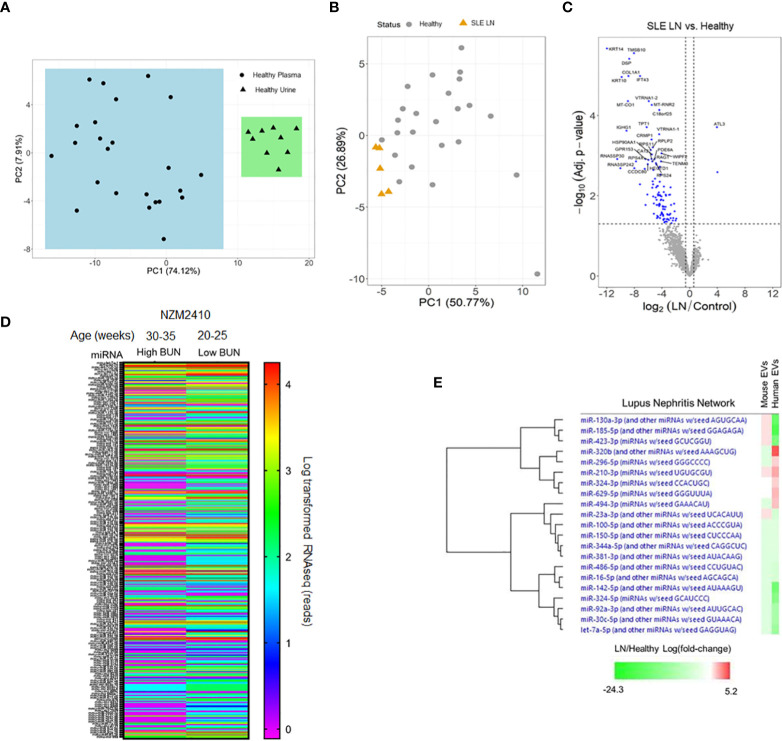
RNA expression profiles from plasma-derived extracellular vesicles (EVs) in human and murine samples. **(A)** EVs were isolated from healthy human urine (N = 10) and plasma (N = 25) sources. **(B)** Plasma-derived EVs were isolated from lupus nephritis (LN) patients (N= 5) and healthy controls (N = 25). **(A, B)** RNA was isolated from EVs for comprehensive, global RNA-sequencing analysis for detectable small RNA sequences. Reads were aligned and principal component analysis was performed to evaluate grouping of the datasets. **(C)** A volcano plot was made from the data generated in **(B)**. **(D)** Plasma was isolated from NZM2410 mice with high and low blood urea nitrogen (BUN) levels (N = 15 per group). EVs were isolated and RNA was purified for sequencing to detect miRNAs. Data from all detected miRNAs (approximately 800) is shown as a heat map and expressed as log-transformed RNAseq reads. **(E)** Fold changes of all log-transformed miRNA expression levels detected in RNA-sequencing of isolated EVs from human (LN/healthy) and murine (high BUN/low BUN) samples were analyzed by pathway analysis software focused on miRNAs associated with LN pathology. Relative fold changes in expression level are shown in a heat map organized by hierarchy.

While exact starting volumes of plasma varied from sample to sample, RNA yields were validated from exosomes isolated from both plasma and urine despite differences in initial volumes; thus providing sufficient samples for multiple library attempts from every isolation. Principal components analysis (PCA) of the annotated small RNA species distinctly separated urine-derived exosomes from plasma-derived exosomes in healthy control samples (**Figure 1A**). These results demonstrate that biological source can differentiate exosome-derived miR profiles. Similarly, PCA of bulk small RNA profiles from plasma exosomes also demonstrated a strong correlation between groups when comparing active LN patients to healthy controls (**Figure 1B**). Furthermore, normalization of expression levels and differential expression corrections yielded significant differences in RNA targets derived from plasma exosomes of active LN patients relative to controls. The greatest differential expression results included vault (v)RNAs (*VTRNA1-1* and *VTRNA1-2*). All vRNA family members share the same chromosomal location (5q31.3) and were each downregulated >20-fold (q-value 8.51E-08; q-value 9.89E-07). The extent and significant differences in bulk RNA signatures are markedly observed when displayed in a volcano plot (**Figure 1C**). The differentiation of body fluid samples by PCA and unique plasma-derived RNA expression profiles indicate that lupus patient exosomes are distinct from healthy controls.

To explore the potential association of EV-encapsulated miRs in lupus-mediated disease progression across species, plasma-derived EVs were isolated from pooled NZM2410 mice using the same ultracentrifugation protocol as the human samples and submitted for RNA-sequencing analysis. When comparing NZM2410 mice with blood urea nitrogen (BUN) levels that we have previously shown to correlate with LN histologically (28), those with lower BUN levels had a significantly discordant EV-encapsulated miR expression profile when displayed as a heat map of approximately 800 miRs (**Figure 1D**). These results indicate that miR expression profiles in exosomes also change in a murine model of lupus in the presence of renal disease. In addition, we also compared the EV-encapsulated miR expression changes associated with LN in mice and humans using pathway analysis software (Ingenuity IPA). After establishing log-transformed miR expression level fold changes associated with disease (LN/healthy and high BUN/low BUN), the data was analyzed to isolate miRs associated with LN pathology. Interestingly, there was substantial overlap with EV-encapsulated miR expression in this pathway when comparing mice and human results by a hierarchical heat map (**Figure 1E**). Collectively, these data demonstrate that EV-encapsulated miR signatures are differentially expressed across species with LN-associated disease progression and similarly regulated in LN-associated pathways in mice and humans.

### 3.2 Extracellular vesicles and EV-encapsulated miRs that bind and activate TLR7 and TLR8 are upregulated in SLE patients with lupus nephritis

**Figure 2:**
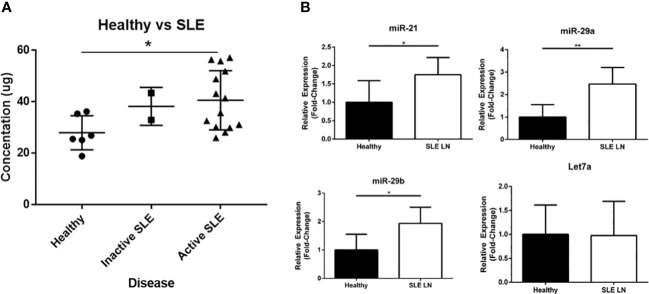
Extracellular vesicles (EVs) and EV-encapsulated miRs that bind and activate TLR7 and TLR8 are upregulated in SLE patients. **(A)** Plasma derived EVs were isolated from healthy volunteers (N = 6) and SLE patients (N = 16; 14 with active disease and 2 inactive) by ultracentrifugation and quantified by an ELISA assay. **(B)** RNA was extracted from EVs isolated from healthy volunteers (N = 6) and active SLE patients (N = 6). Expression of miR-21, miR29a, miR-29b, and Let7a was measured by RT-PCR analysis. Data was normalized to RNU-44 internal control expression and shown as a relative fold-change. Values are the mean ± SEM with indicated p values calculated via paired, two-tailed, Student’s t tests. *p ≤ 0.05; **p ≤ 0.01.

To further explore the association of exosome-encapsulated RNA biocargo and disease activity, we isolated plasma from SLE patients (including inactive/active and with/without renal involvement) and compared exosome concentrations to age/sex-matched healthy controls by ELISA. Using the well-characterized CD9 expression biomarker to immobilize exosomes from plasma, our results showed that SLE exosome levels were higher than healthy controls both with and without active disease or renal involvement (**Figure 2A**). Specifically, exosome concentrations of active SLE patients were 2-fold greater when compared to healthy controls and statistically significant. Since exosome concentrations are higher in SLE on average, normalized RNA values will not physiologically reflect relative levels when comparing SLE to healthy control samples. Consequently, while the exosome-derived small RNA profiling above was valuable to establish differential content, individual target expression levels from total RNA are needed to evaluate potential functional impact *in vivo*. To this accord, specific exosome-derived miR targets were selected based on published data and included miR-21 (22), miR-29a (22), and miR-29b (33); each has been shown to be packaged into exosomes and signal through recipient cells via TLR7 or TLR8. While no difference was observed in Let7a, expression of miR-21, miR-29a, and miR-29b was significantly elevated in active SLE patients with LN relative to age/sex-matched healthy controls (**Figure 2B**). The data was normalized to RNU-44, as this has been shown to be the most stably expressed endogenous control in EVs and was specifically included in a universal method for miR RT-qPCR data normalization (34, 35). Considering that these exosome-encapsulated miRs can activate TLR7 or TLR8 and that immunomodulatory exosomes are upregulated in SLE, combinational antagonism of miR-21, miR-29a, and miR-29b represents a novel therapeutic strategy to evaluate.

### 3.3 RNA sequencing of PBMCs reveals estrogen-mediated upregulation of miR processing machinery

Studies examining intracellular miR expression in PBMCs of patients with SLE have identified distinct disease-associated changes (36). Furthermore, our previous work has demonstrated that estrogen can significantly upregulate both TLR7 and TLR8 expression and lower the threshold of immune cell activation more extensively in females (20). To examine the potential effects of estrogen on miR processing and production, human PBMCs isolated from whole blood were cultured in hormone free conditions overnight before stimulation with 10 nM of 17β-estradiol (estrogen; E2). Total RNA was isolated from cell lysates and RNA libraries were prepared for RNAseq; E2-treated samples were compared and normalized to untreated controls for each individual donor. Comprehensive signaling and network pathway analysis of RNAseq data predicted upregulation of estrogen-dependent breast cancer signaling by canonical pathway overlay and estrogen receptor upregulation by overlap upstream analysis (data not shown). Additionally, further overlay analysis identified upregulated pathways that included several involved in miR transcription/processing (**Supplementary Figure 3A**). Specifically, argonaute-2 (AGO2) showed an IPA overlap p-value of 0.006, which resulted from upregulation of AGO2 mRNA expression by 1.3-fold with E2 treatment and significant downregulation of FOS, miR-127, miR-34, and miR-27. Moreover, Dicer1 mRNA (a Ribonuclease III enzyme) was stimulated 1.3-fold with estrogen treatment and an IPA overlap p-value of 0.001 was observed that resulted from miR-34, miR-196, and miR-218 suppression. Estrogen-induced mRNA expression in expected genes based on our previous studies as well as other genes involved in intracellular miR processing (**Supplementary Figure 3B**). Specifically, Drosha RNase III mRNA was induced 1.2-fold with estrogen treatment and several members of the RNA polymerase III family (POLR3A, POLR3D, and POLR3D) were upregulated 1.3-fold, 1.4-fold, and 1.3-fold, respectively. In concordance with our previous studies (20, 25, 37), estrogen-mediated upregulation of STAT1 (1.4-fold), ESR1 (1.3-fold), ESR2 (1.2-fold), TLR8 (1.5-fold), STAT4 (1.3-fold), and TLR7 (1.3-fold) were also demonstrated. These data in combination with our previous work suggest that E2 may play a role in enhanced cellular miR production and non-canonical activation of overexpressed TLR7 and TLR8 in SLE patients via exosome-mediated signaling.

### 3.4 Inhibition of inflammation and disease progression in human-mouse chimeras by antagonizing miRs that bind TLR7 and TLR8

Our previous data has shown that liposome-encapsulated miR-21 can indeed signal through TLR8 to induce TLR8 expression, pro-inflammatory cytokine signaling, and additional EV secretion in human macrophages and primary PBMC cultures (37). Furthermore, we have also demonstrated that locked nucleic acid (LNA) modification can be used as an antagonist to block the induction of TLR8 expression by endogenous EVs (19). To block exosome-encapsulated, miR-induced inflammation via miR-21, miR-29a, and miR-29b, human PBMCs derived from active LN patients were treated with a cocktail of LNA miR antagonists (LNA cocktail) or equimolar of LNA control (miRScramble) prior to adoptive transfer into immunodeficient murine recipients. While *ex vivo* LNA treatment is less optimal and translatable to clinical applications, exploratory preclinical studies with these approaches necessitate this protocol due to the limitations and considerations associated with *in vivo* LNA manufacturing, biodistribution, PK/PD, and efficient of delivery to PBMCs. LNAs contain a modified ribose moiety and were used here to antagonize miR functionality *in vivo*. They are significantly more resistant to enzymatic degradation than RNA and have been shown to persist *in vivo* for over a week (38). Adoptively transferred PBMCs were engrafted into immunodeficient mouse recipients for three weeks; our previous work recapitulating autoimmune disease in the NSG mouse model demonstrated that this period of time was optimal for histopathological observation of target organ inflammation without confounding graft versus host reaction (30). A humanized mouse approach was chosen for this study over the well-established transgenic mouse models of lupus for several reasons. First, while there is homology in miR sequences and expression between mice and humans, the sequences, expression, identity and affinity of mRNA targets, and post-transcriptional regulation are not identical (39), which would make the results less applicable to human translation. In addition, mouse and human TLR8 have distinct functionality and receptor specificities, as shown by studies examining affinity and activation to natural and synthetic TLR8 ligands (40, 41). Human PBMC engraftment to establish human-mouse chimeras in immunodeficient murine backgrounds all have associated limitations and considerations (42). Consequently, both NRG and NSG immunodeficient mice were used and demonstrated similar results in this study. Successful PBMC engraftment was observed in NRG and NSG mice and with both LNA treatment conditions, as demonstrated by similar levels human T-cells (CD4+ and CD8+), B-cells, monocytes, and NK cells recovered from whole blood of all chimeric mice (**Supplementary Figure 4**). Although similar levels of PBMC engraftment were observed, disease progression was indicated in mice treated with LNA control; while weights of uninjected immunodeficient mice were similar trending longitudinally to LNA cocktail, LNA control-treated mice showed a notable loss of body mass at the three week timepoint (**Supplementary Figure 5**).

**Figure 3:**
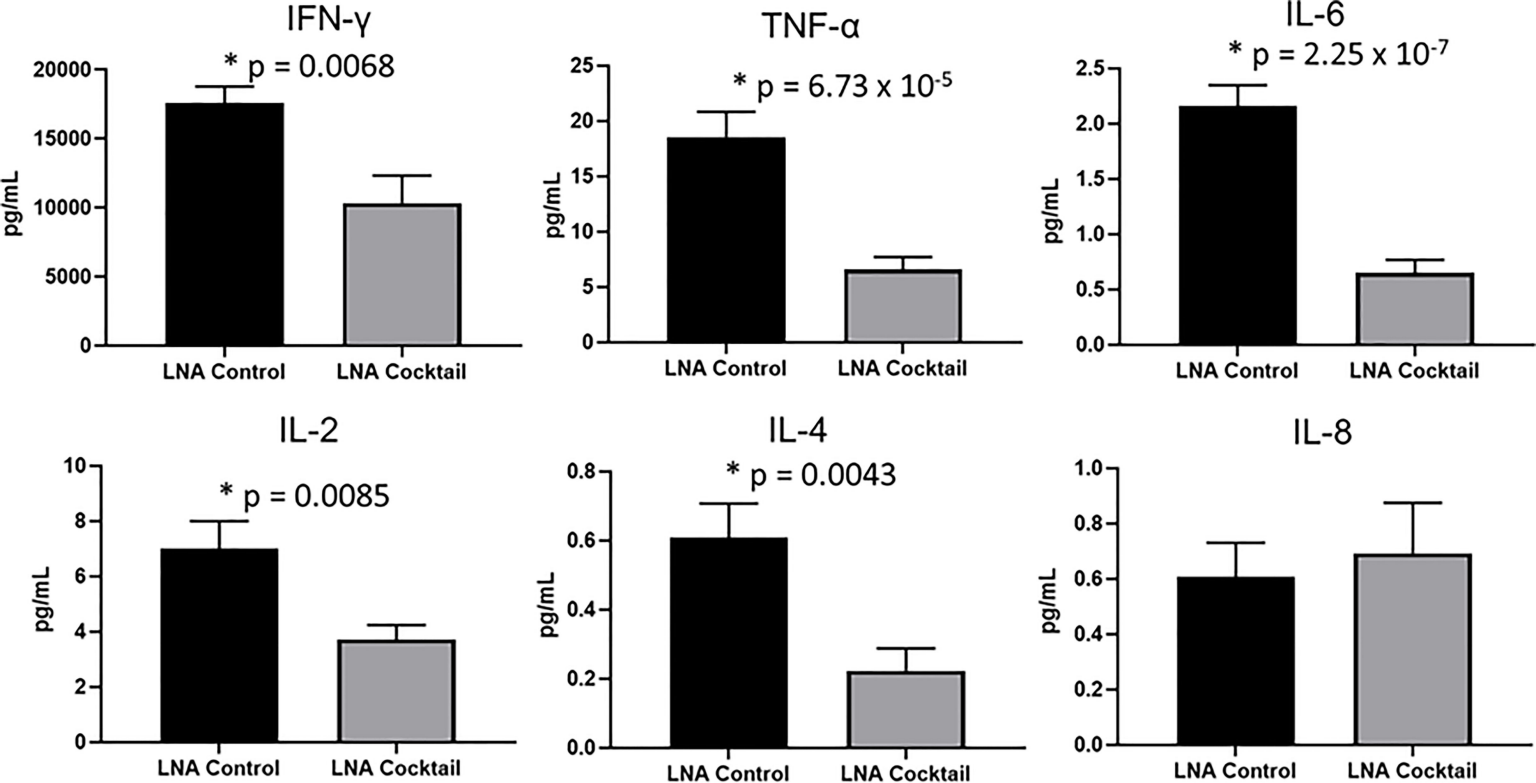
Proinflammatory cytokine expression is reduced in chimeric mice treated with miR antagonists. PBMCs from active SLE patients (N = 4) were transfected *ex vivo* with a cocktail of locked nucleic acid miR antagonists (LNA cocktail) targeting miR-21, miR-29a, and miR-29b, or a nonsense control (LNA control). Serum proinflammatory cytokine expression was measured in chimeric mice (N = 10) after 21 days by ELISA. Values are the mean ± SEM with indicated p values calculated via paired, two-tailed, Student’s t tests. *All values of p ≤ 0.05 considered statistically significant.

**Figure 4:**
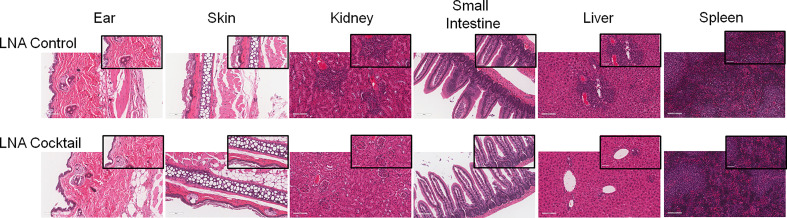
Inhibition of target organ inflammation by antagonizing miRs that bind TLR7 and TLR8 suppresses inflammation in human-mouse chimeras. PBMCs from active SLE patients were adoptively transferred into immunodeficient mice to produce chimeras. For each SLE patient (N = 4), 2 or 3 immunodeficient mice were used, depending on cell yield (N = 10 total). To block miR-induced inflammation, PBMCs were transfected *ex vivo* with a cocktail of locked nucleic acid miR antagonists (LNA cocktail) targeting miR-21, miR-29a, and miR-29b, or a nonsense control (LNA control) prior to injection. 21 days after adoptive transfer, tissues were collected and processed for H&E staining. Large panel images taken at 20X magnification (scale bar representing 100 µm) and insets taken at 40X (scale bar representing 50 µm).

**Figure 5:**
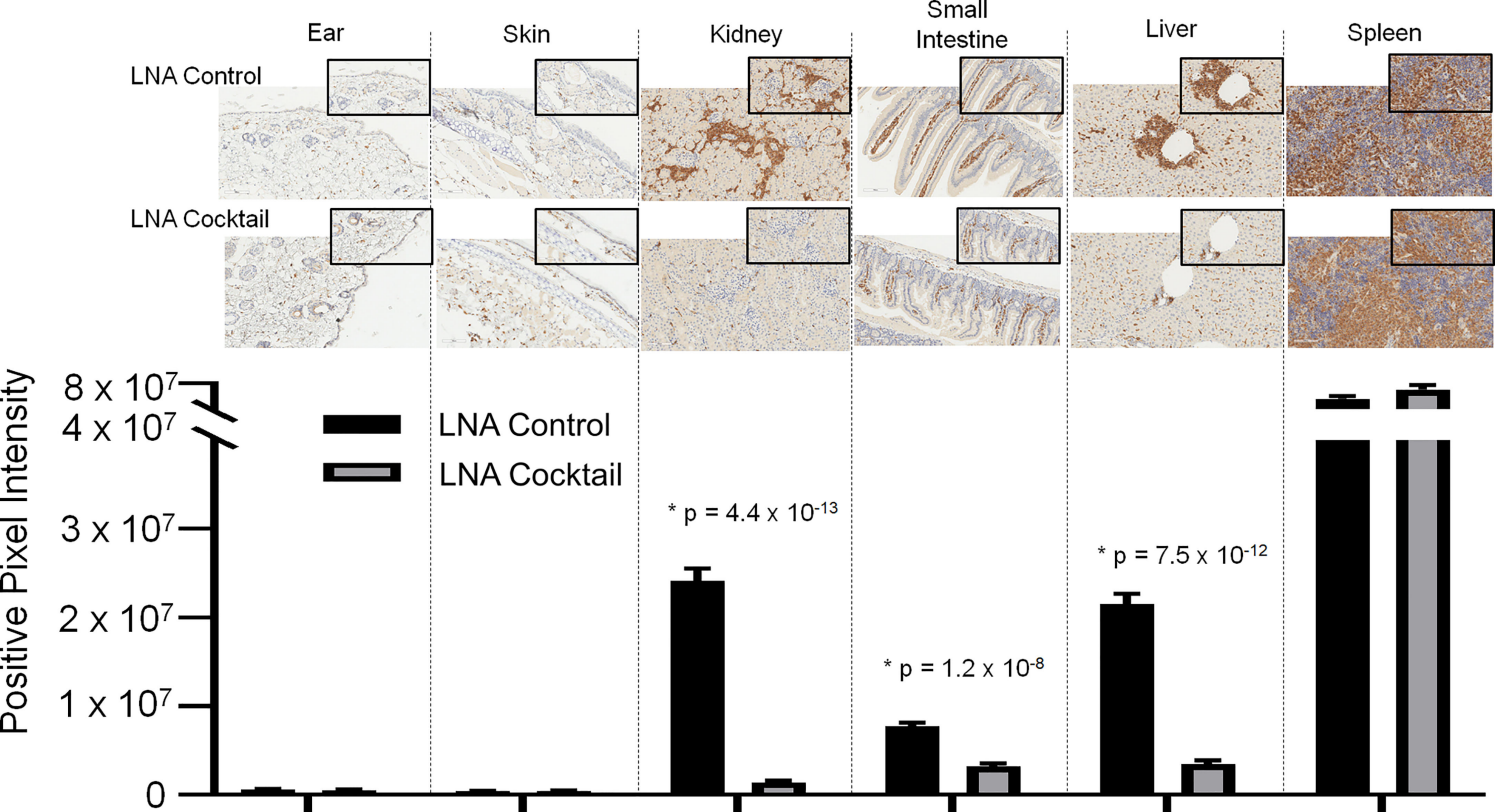
Immunohistochemical analysis of the infiltrates from chimeric mice confirm the presence of human CD3+ T cells in target organs. Target organ inflammation was measured in chimeric mice by IHC for human CD3 detection to compare the effects of a cocktail of locked nucleic acid miR antagonists (LNA cocktail) targeting miR-21, miR-29a, and miR-29b to a nonsense control treatment (LNA control). Large panel images taken at 20X magnification (scale bar representing 100 µm) and insets taken at 40X (scale bar representing 50 µm). Histopathological assessment was performed as described in the materials and methods by digital analysis of scanned slides to evaluate relative CD3 expression. Values are the mean ± SEM. Single t test comparisons were made in addition to two-way ANOVA analysis. Results of single t tests comparing control to cocktail in each individual tissue with indicated p values are shown. *All values of p ≤ 0.05 considered statistically significant.

To assess the effects of antagonizing miR-21, miR-29a, and miR-29b with LNA inhibitors for comparison to LNA control, serum and tissue samples were collected from NSG and NRG mice. Serum was analyzed for proinflammatory cytokine expression to measure the systemic response at three weeks post-adoptive transfer. While no differences were observed in IL-8 expression, treatment with the LNA inhibitor cocktail targeting miR-21, miR-29a, and miR-29b resulted in significant decreases in IFN-γ by 40% (p = 0.0068), TNF-α by 67% (p = 6.73 x 10-5), IL-6 by 70% (p = 2.25 x 10-7), IL-2 by 47% (p = 0.0085), and IL-4 by 63% (p = 0.0043) relative to PBMCs treated with LNA controls (**Figure 3**). While little to no inflammation was observed in the skin and ear, histopathological examination by H&E showed a robust inflammatory response in the small intestine, liver, and kidney with control treatment, which was markedly reduced with miR inhibition via LNA cocktail treatment (**Figure 4**). Specifically, renal inflammation was characterized by infiltrates into the glomerulus leading to abnormal histological architecture and a loss of observable capsular (Bowman’s) space. Hepatic inflammatory responses were largely observed in the portal triad area of tissue sections encompassing the portal vein, hepatic artery, and bile ductule. Also, histopathological evaluation of intestinal infiltrates demonstrated more robust infiltration of the lamina propria and submucosa in LNA control mice. To show that the inflammation was resulting from engrafted human PBMCs and to quantitate the extent of response for statistical comparison, IHC was performed with digital histopathological image analysis to detect and quantify human CD3+ T cells present in the tissue sections. Since our previous work engrafting PBMCs from patients with autoimmune disease into immunodeficient mouse recipients showed that target organ inflammation was comprised of CD4+ and CD8+ T cells (30), IHC for CD3 was used here to analyze both immune cell subtypes collectively. Nonparametric analysis by single t tests individually demonstrated statistically significant reductions in CD3+ human T cell infiltrates in the kidney by 17-fold (p = 4.4 x 10-13), the small intestine by 2.4-fold (p = 1.2 x 10-8), and the liver by 6.2 fold (p = 7.5 x 10-12) with LNA-mediated inhibition of miR-21, miR-29a, and miR-29b (**Figure 5**). Similarly, results comparing across tissue type (p < 0.0001), LNA treatment (p = 0.0023), and both (p = 0.0007) were all statistically significant by two-way ANOVA analysis as well. Spleens of all mice showed high levels of CD3+ human T cell detection, which is indicative of successful human immune cell engraftment and reconstitution *in vivo*. These results establish feasibility of this chimeric platform to study LNA-based therapeutics to control autoimmune-mediated inflammatory responses and indicate that inhibition of miR-21, miR-29a, and miR-29b can suppress disease pathology.

## 4 Discussion

**Figure 6:**
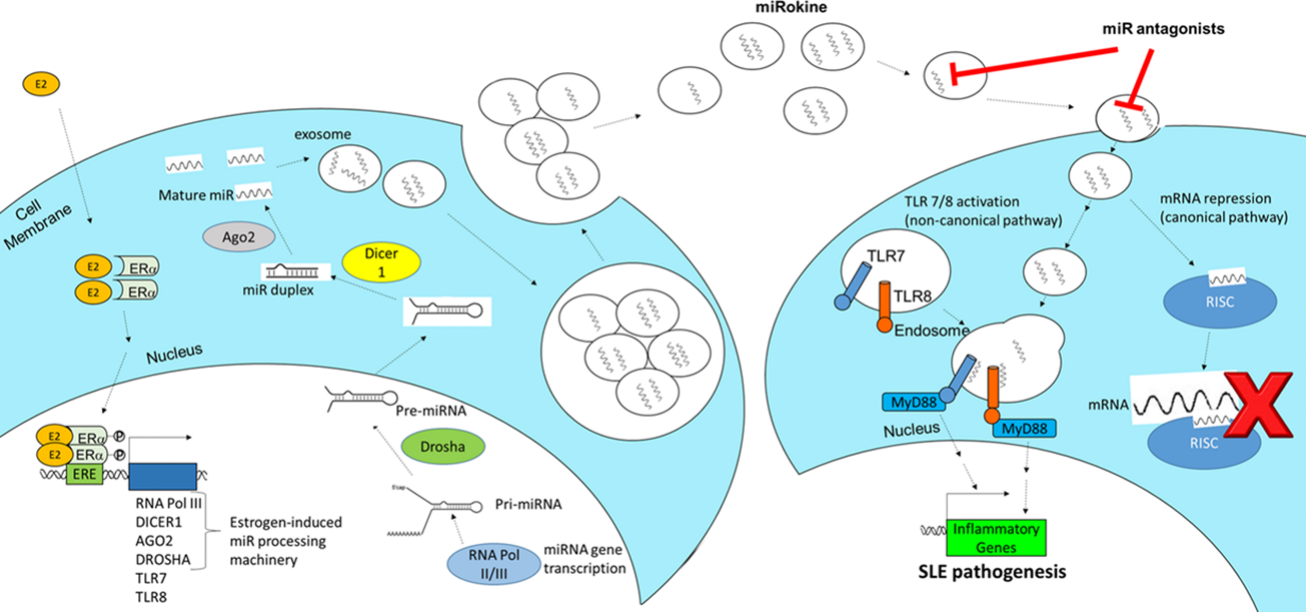
Schematic of the proposed mechanism of estrogen-mediated miR production and inflammation via extracellular vesicle signaling in SLE. Estrogen (E2) enters an immune cell, dimerizes with estrogen receptor (ER)α, translocates to the nucleus, and promotes the expression of miRNA (miR) processing machinery, including RNA polymerase III, Dicer1, AGO2, and Drosha. These miRs are packaged and secreted in EVs, which are taken up by recipient immune cells. When EV-derived miR cargo is taken up, it can function to regulate gene expression in the recipient cell via two pathways. The canonical pathway involves miRs binding to target mRNAs through the RNA-induced silencing complex (RISC). In the non-canonical pathway, EV-encapsulated miRs fuse with endosomes and bind to TRL7 or TLR8 to stimulate proinflammatory gene expression and additional EV secretion, which promotes SLE pathogenesis.

Elucidating the pathogenesis and molecular mechanisms driving inflammatory responses in SLE can yield desperately needed diagnostics and targeted therapies. Despite 60 years of research, only 3 new agents (belimumab, anifrolumab-fnia, and voclosporin) have been approved as treatments for this autoimmune disease in recent years. Similarly, clinical tests to diagnose SLE or to predict flares have also lagged behind other scientific advances in medicine. In the “omics” era of biomarker discovery via DNA/RNA sequencing, epigenetics, and proteomics, SLE is still largely diagnosed and treated according to clinical classification criteria (43, 44). Typical laboratory testing conventionally ordered in the clinic to aid in SLE diagnosis includes anti-nuclear antibodies (ANA), anti-double-stranded DNA antibodies (anti-dsDNA), and complement levels, but these tests have limited sensitivity and specificity (45–47). While the gold standard for LN diagnosis is a kidney biopsy to confirm renal involvement, this procedure is invasive and has associated risks and complications. In this study, we identify a novel exosome-mediated miR signaling pathway that is associated with SLE disease activity and demonstrate that blocking upregulated miRs in exosomes can inhibit the pathogenic inflammatory response. SLE-associated miRs packaged in exosomes can potentially be developed as clinical/companion diagnostics and miR antagonists can be a novel area of future therapeutic development. Collectively, the results from this study suggest a putative mechanism of estrogen-mediated miR synthesis contributing to exosome-encapsulated miR signaling and induction of inflammatory responses in SLE by signaling through TLR7 or TLR8 (**Figure 6**). In this pathway, estrogen stimulates the production of miRs by inducing the expression of RNA polymerase III, Dicer1, AGO2, and Drosha. Intracellular miRs are packaged and secreted in exosomes for delivery to recipient cells. The miRs within exosomes act as cytokines to mediate cell-to-cell communication in the inflammatory response; following this nomenclature, we have previously referred to exosome-encapsulated miRs as miRokines (37). Upon entry into the new cell, miRokines can function in the canonical pathway by associating with RNA-induced silencing complexes (RISC) to bind complementary mRNAs and suppress gene expression or non-canonically by binding to TRL7 or TLR8. Activation of TLR7 and/or TLR8 by miR ligands can stimulate additional exosome secretion and proinflammatory gene expression, which can contribute to SLE pathogenesis.

Estrogen has a pleotropic effect on immune system function and can significantly stimulate macrophage and lymphocyte proliferation (48). Our previous studies have shown that estrogen can lower the threshold of immune cell activation and that this response is more robust in females (20). These data suggested that estrogen may enhance immune system activation, which could augment infectious disease responses, but also may contribute to autoimmune disease development and pathogenesis. In SLE, disease-associated sex-bias is evident (49), as most clearly demonstrated by the patient population; the adult premenopausal female to male ratio of SLE is 9:1, which then becomes closer to 2:1 either during childhood or post menopause (50). Here, we made a preliminary observation that estrogen may stimulate the expression of proteins involved in intracellular miR processing. While a direct correlation of estrogen and exosome production has not been established, previous studies have demonstrated that estrogen can have a significant impact on the intracellular regulation of miR expression. Estrogen receptor (ER)α positive and hormone responsive breast cancer cell lines (MCF-7 and ZR-75.1) differentially regulated 172 miRs with estrogen treatment (51). Moreover, unique miR expression profiles have been reported in human breast cancer and these differences correlated with expression of ER, tumor stage, number of positive lymph nodes, and vascular invasion [reviewed in (52)]. Our results suggest that estrogen stimulation of primary human PBMCs can upregulate miR processing machinery, which may lead to the unique intracellular miR profiles observed in other studies and account for the distinct miRs packaged within exosomes of SLE patients. Additional studies are needed to explore this novel inflammatory mechanism.

Our data characterize EVs and the associated RNA biocargo in addition to evaluating the therapeutic strategy of miR inhibition in SLE. While the delivery of RNA biocargo within EVs to influence recipient cell function has only recently been explored therapeutically, the clinical success of SARS-CoV-2 mRNA vaccines has validated this as a biologically relevant and viable therapeutic strategy. Both the mRNA-1273 and BNT162b2 mRNA-based vaccines for SARS-CoV-2 are packaged into lipid nanoparticles (LNPs) that are approximately 100 nm in size (53). Similarly, both the liposomes used here to deliver LNA antagonists and the exosomes used for RNA sequencing analysis from plasma samples were also approximately 100 nm in size. While blocking RNA expression is a relatively new therapeutic strategy, there have been four small interfering (si)RNA drugs approved by the FDA to date (54). LNAs represent the third generation of chemically modified RNA therapeutics and exhibit binding affinities and RNA neutralization capacities far superior to siRNA (55). In addition to enhanced affinity, potency, and specificity to RNA targets, LNAs also exhibit bioavailability and stability, as indicated by the diverse tissue distribution and long half-life *in vivo* observed in the clinical trials of a miR-122 LNA antagonist (miraversen) (56). Considering the preclinical and clinical development of RNA targeting and delivery through LNPs similar in size to exosomes, our data suggests that exosome-encapsulated RNAs are biologically significant and represent a novel pharmacological platform to evaluate therapeutically in SLE.

Despite the biological relevance of exosomal biocargo signaling in regulating inflammatory processes and the potential of exosomes to be used as disease-associated biomarkers, very little data has been generated to date in the study of autoimmune disease. While distinct miR signatures have been identified in exosomes derived from LN patients and specific miRs have been associated with renal fibrosis and disease activity (18, 57, 58), very few studies have evaluated blood derived exosomes in SLE. Moreover, although these studies of exosomes in SLE blood circulation identified differential regulation of specific miRs (miR-145a, miR-21, and miR-155), the EVs evaluated and data generated may be physiologically irrelevant considering that serum sources are subject to platelet-derived exosome secretion/contamination while the blood sample is clotting during experimental processing (59). As predicted, our results demonstrated distinct EV-associated miR profiles from blood and urine. Unlike microvesicles or apoptotic bodies that shed directly from the cell membrane, exosomes contain distinct biocargo, closely correlating with the inner and surface contents of their cells of origin. Accordingly, previous genomic and proteomic analyses have demonstrated that cellular content and exosomes from those cells contain significant overlap (60–62). Our data also show that plasma-derived EVs are upregulated in both inactive and active SLE patients and contain distinct small RNA profiles. This data is in concordance with the well-established correlation of exosome secretion to inflammatory diseases and tumor-associated inflammation [reviewed in (63)]. Interestingly, vault (v)RNAs (*VTRNA1-1 and VTRNA1-2*) were significantly downregulated in active LN patients. vRNAs have been detected in exosomes previously and are thought to play a role in intracellular and nucleocytoplasmic transport processes (64). Furthermore, previous research has shown that vRNAs can confer resistance to autophagy when overexpressed (65, 66) as well as inactivate protein kinase R signaling, which inhibits interferon responses (67). Consequently, these findings suggest that exosome delivered vRNAs could have significant influence over the cellular regulation of autophagy (68) and interferon signaling (69) that is observed in patients with active LN.

In summary, the results of this study demonstrate dysregulated small RNA expression patterns in EVs of SLE patients that potentially can be targeted therapeutically to suppress disease pathogenesis. Future work will be directed toward defining the precise mechanistic role of estrogen in this process, examining the candidacy of exosomes as disease-associated diagnostic biomarkers, and identifying additional miRs to target therapeutically. In addition to miRs, exosome biocargo includes surface-expressed and intra-exosomal protein, mRNA, vRNA, and long noncoding (ln)RNA. The roles of these additional RNA species in cell-to-cell communication and as putative biomarkers have yet to be explored in SLE. Furthermore, biomarker characterization of exosome protein expression can define cell of origin, which may be used to predict specific organ involvement early in the disease course. Previous studies have also demonstrated that exosomes can contain cytokines (70); however, the biological relevance of this observation and the potential role that this may play in immunopathology has not been described. Through these follow-up studies, the roles of different exosomal biocargo in the diagnosis and treatment of SLE will be better characterized.

## Data availability statement

The original contributions presented in the study are publicly available. This data can be found here: https://figshare.com/projects/2024_Jarjour_SLE_exosome_miRs/203685.

## Ethics statement

The studies involving human participants were reviewed and approved by The Ohio State University Wexner Medical Center Institutional Review board (IRB); Protocol Numbers: 2011H0094, The Ohio State University Rheumatology and Immunology Lupus Clinic Registry; and 2015H0340, Multi-Source Examinations of Biomarkers in Autoimmune Diseases. The patients/participants provided their written informed consent to participate in this study. The animal study was reviewed and approved by The Ohio State University Wexner Medical Center, Institutional Animal Care and Use Committee and University Laboratory Animal Resources (protocol 2017A00000032).

## Author contributions

Conceived and designed the experiments of the study: (NY, ER, WJ). Data collection and analysis: (NY, ES, KJ, ER, WJ). Performed experiments: (NY, ES, BZ, SB, KJ, ER). Edited manuscript: (NY, ES, KJ, LW, ER, WJ). Statistical assessments: (NY, ER). Wrote manuscript: (NY, ER, WJ). Contributed reagents/materials/analysis tools: (LW, ER, WJ). Made substantial, direct and intellectual contribution to the work, and approved it for publication: (NY, ES, KJ, BZ, SB, LW, ER, WJ).

## Supplementary material

Supplementary Figure 1 Extracellular vesicle (EV) characterization demonstrates representative exosome size and enriched miRNA fractions. **(A)** Following EV isolation by ultracentrifugation, biological samples were analyzed by nanoparticle tracking (representative samples). **(B)** Representative bioanalyzer analyses showing that the small RNA fraction is detectable from EV isolations of both urine and plasma samples derived from LN patients and healthy subject samples. Arrows indicate small RNA (lncRNA and miRNA). **(C)** Representative results from bioanalyzer analysis of 10 mL human plasma. A sharp peak at 10 nt is the positive control and is followed by the isolated small RNA peak ranging approximately from 20 nt to 200 nt (virtual, computer generated RNA electrophoresis to the right of the histogram).

Supplementary Figure 2 Extracellular vesicle (EV) RNA isolations are plasma input-dependent and yield products with predicted exosome size and surface marker expression. **(A)** EVs were isolated from various plasma volumes and produced a trend of larger RNA yields with increased plasma volumes. Bioanlayzer readouts with each plasma volume are shown below along with a reference control ladder. **(B)** Biological samples were analyzed by nanoparticle tracking analysis following EV isolation of 10 mL or 20 mL of plasma by ultracentrifugation. The average peak is approximately 110 nm, which is within predicted exome size ranges of 50 - 150 nm in diameter. **(C)** Protein concentrations of EV isolations are increased with longer ultracentrifugation time and correlate with enhanced detection of exosome biomarker CD63 by Western Blotting.

Supplementary Figure 3 Estrogen induces intracellular miR processing pathways in human PBMCs. Human PBMCs were isolated from healthy volunteers (N = 6) and stimulated with 17β-estradiol (estrogen; E2). RNA was isolated for RNA-sequencing analysis for detectable miR and mRNA sequences. Reads were aligned and analyzed by Ingenuity Pathway Analysis tools examining comprehensive effects of mRNA and miR expression on cellular signaling pathway activation. **(A)** Overlapping p-values from Ingenuity analysis are indicated for select proteins involved in miR synthesis. **(B)** RNA-sequencing read hits are indicated for miR synthesis pathway mRNAs and other known estrogen-regulated genes. *All values of p ≤ 0.05 considered statistically significant.

Supplementary Figure 4 Reconstitution of human PBMCs is observed with control or miR inhibition. Whole blood was analyzed by flow cytometry in chimeric mice following treatment with LNA cocktail or LNA control. Immune cell subtype distribution was measured using the indicated markers, as detailed in the methods section.

Supplementary Figure 5 Expected weight-loss prevented with inhibition of miR targets in humanized mice. Weights were recorded over time in chimeric mice treated with locked nucleic acid miR antagonists (LNA cocktail) targeting miR-21, miR-29a, and miR-29b for comparison to a nonsense control treatment (LNA control).
