# Supplementary figures and images for: Inhibition of miRNA associated with a disease-specific signature and secreted via extracellular vesicles of systemic lupus erythematosus patients suppresses target organ inflammation in a humanized mouse model
Source: Front Immunol. 2024 Jun 13;14:1090177. doi: 10.3389/fimmu.2023.1090177 (PMC11208704; doi:10.3389/fimmu.2023.1090177)

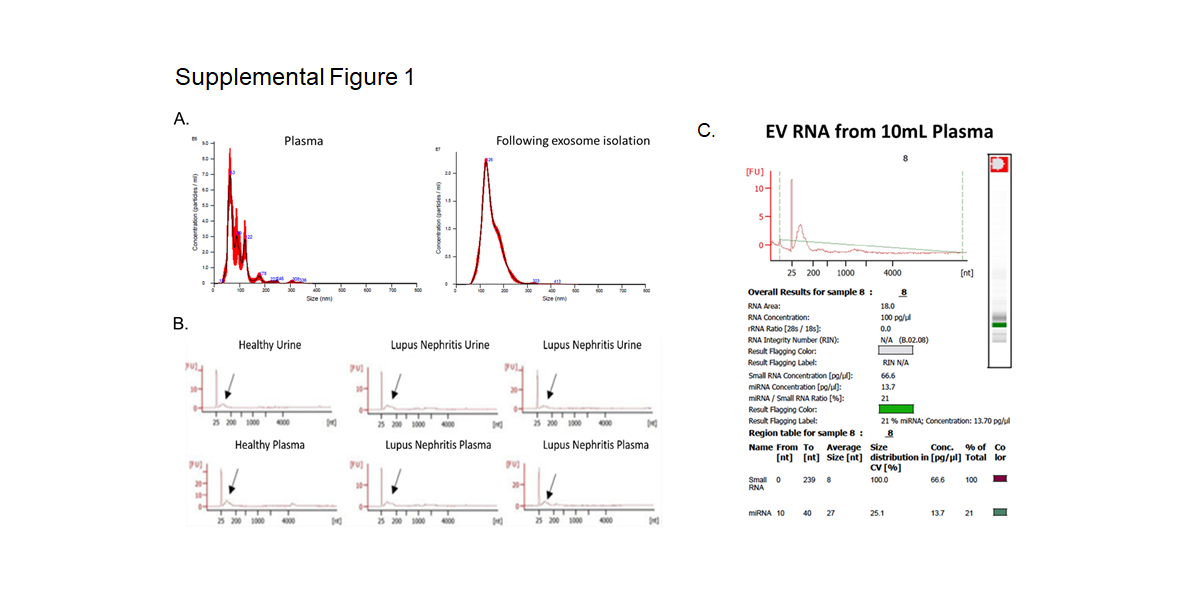

Supplement: Supplementary Figure 1 — Extracellular vesicle (EV) characterization demonstrates representative exosome size and enriched miRNA fractions. (A) Following EV isolation by ultracentrifugation, biological samples were analyzed by nanoparticle tracking (representative samples). (B) Representative bioanalyzer analyses showing that the small RNA fraction is detectable from EV isolations of both urine and plasma samples derived from LN patients and healthy subject samples. Arrows indicate small RNA (lncRNA and miRNA). (C) Representative results from bioanalyzer analysis of 10 mL human plasma. A sharp peak at 10 nt is the positive control and is followed by the isolated small RNA peak ranging approximately from 20 nt to 200 nt (virtual, computer generated RNA electrophoresis to the right of the histogram). [file Image_1.tif]

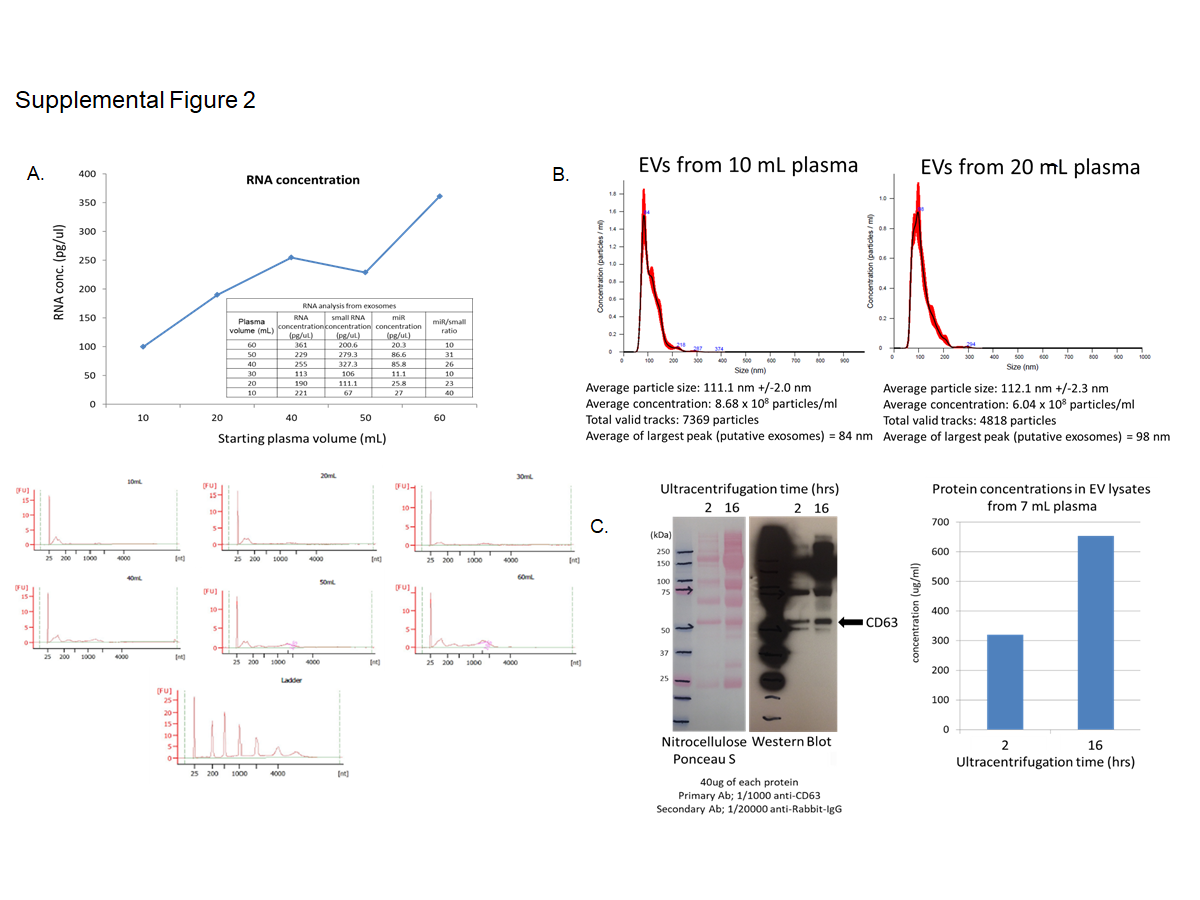

Supplement: Supplementary Figure 2 — Extracellular vesicle (EV) RNA isolations are plasma input-dependent and yield products with predicted exosome size and surface marker expression. (A) EVs were isolated from various plasma volumes and produced a trend of larger RNA yields with increased plasma volumes. Bioanlayzer readouts with each plasma volume are shown below along with a reference control ladder. (B) Biological samples were analyzed by nanoparticle tracking analysis following EV isolation of 10 mL or 20 mL of plasma by ultracentrifugation. The average peak is approximately 110 nm, which is within predicted exome size ranges of 50 - 150 nm in diameter. (C) Protein concentrations of EV isolations are increased with longer ultracentrifugation time and correlate with enhanced detection of exosome biomarker CD63 by Western Blotting. [file Image_2.tif]

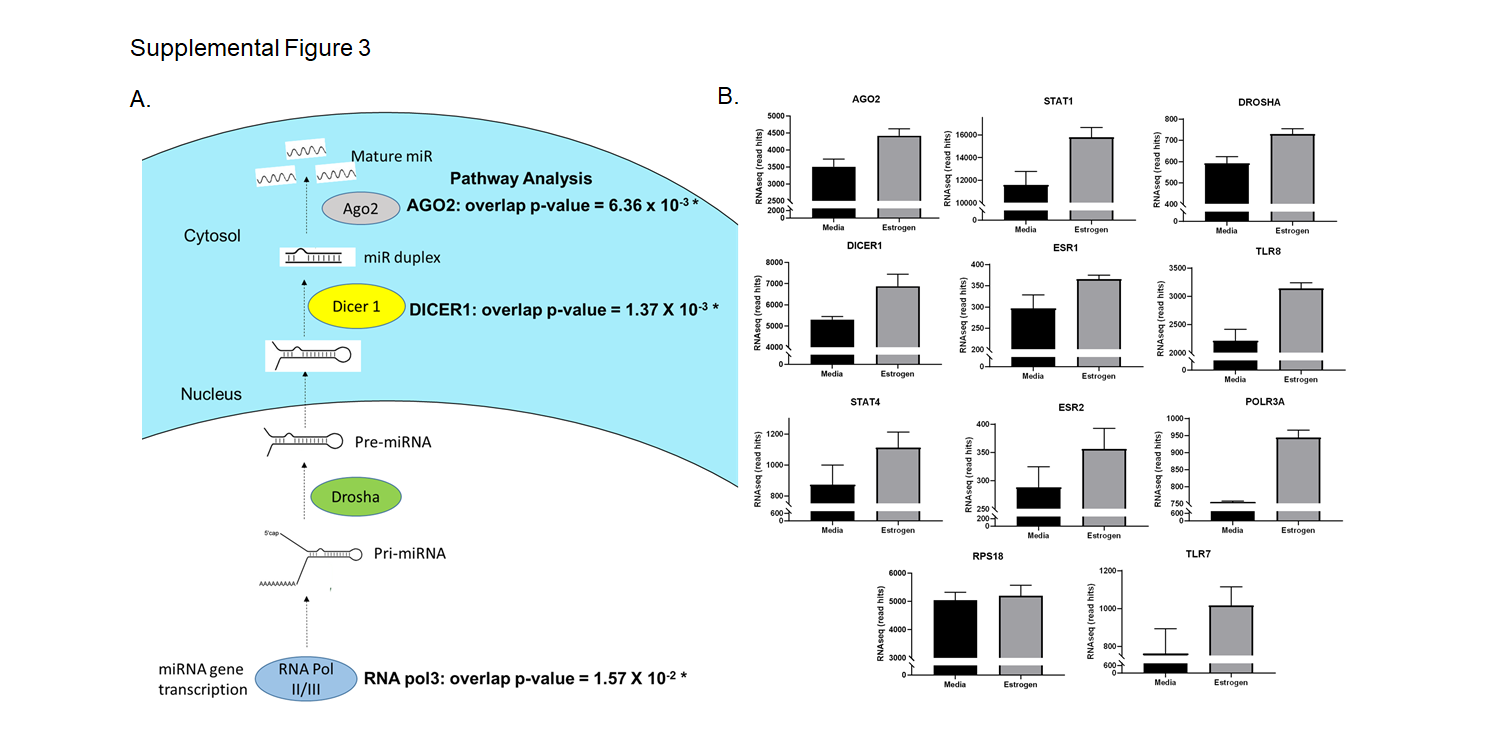

Supplement: Supplementary Figure 3 — Estrogen induces intracellular miR processing pathways in human PBMCs. Human PBMCs were isolated from healthy volunteers (N = 6) and stimulated with 17β-estradiol (estrogen; E2). RNA was isolated for RNA-sequencing analysis for detectable miR and mRNA sequences. Reads were aligned and analyzed by Ingenuity Pathway Analysis tools examining comprehensive effects of mRNA and miR expression on cellular signaling pathway activation. (A) Overlapping p-values from Ingenuity analysis are indicated for select proteins involved in miR synthesis. (B) RNA-sequencing read hits are indicated for miR synthesis pathway mRNAs and other known estrogen-regulated genes. *All values of p ≤ 0.05 considered statistically significant. [file Image_3.tif]

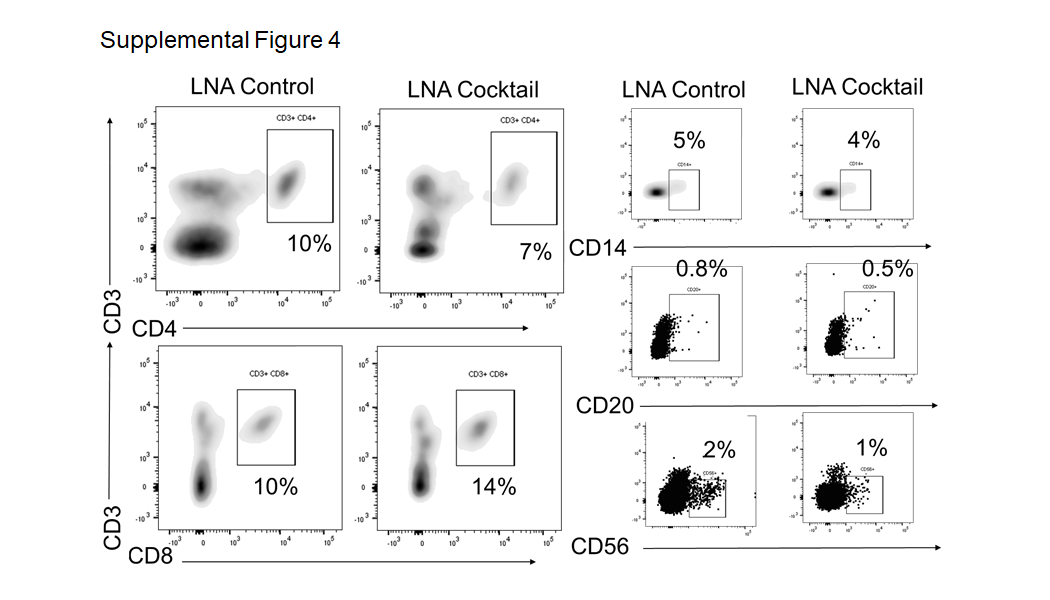

Supplement: Supplementary Figure 4 — Reconstitution of human PBMCs is observed with control or miR inhibition. Whole blood was analyzed by flow cytometry in chimeric mice following treatment with LNA cocktail or LNA control. Immune cell subtype distribution was measured using the indicated markers, as detailed in the methods section. [file Image_4.tif]

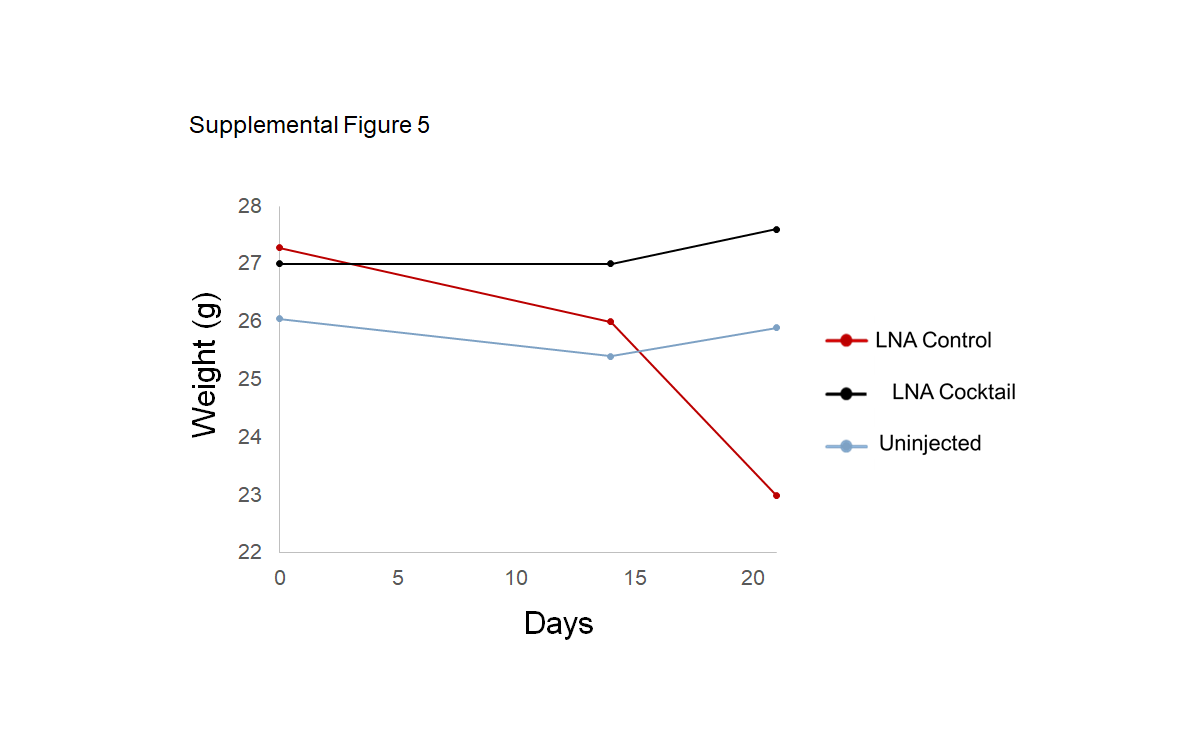

Supplement: Supplementary Figure 5 — Expected weight-loss prevented with inhibition of miR targets in humanized mice. Weights were recorded over time in chimeric mice treated with locked nucleic acid miR antagonists (LNA cocktail) targeting miR-21, miR-29a, and miR-29b for comparison to a nonsense control treatment (LNA control). [file Image_5.tif]
